# Potent immunogenicity in *BRCA1*‐mutated patients with high‐grade serous ovarian carcinoma

**DOI:** 10.1111/jcmm.13678

**Published:** 2018-05-31

**Authors:** Ying Dai, Chengdu Sun, Yi Feng, Qingzhu Jia, Bo Zhu

**Affiliations:** ^1^ Institute of Cancer Xinqiao Hospital Third Military Medical University Chongqing China

**Keywords:** BRCA, high‐grade serous ovarian carcinomas, immune checkpoint inhibitor, TCGA, total tumour burden, tumour microenvironment

## Abstract

High‐grade serous ovarian carcinomas (HGSOCs) were among the tumours with an unsatisfactory outcome of immune checkpoint inhibitors (ICIs). It is imperative to develop feasible biomarker for identifying responsive candidates and guiding precise immunotherapy for HGSOC patients. Here, we analysed genomic data of patients with HGSOCs to depict their immunological phenotype of tumour microenvironment (TME) and figure out the major determinants of immunogenicity. In comparison with other solid tumours, we observed the lowest levels of PD‐L1, total mutation burden (TMB) and cytolytic molecules in HGSOCs. Surprisingly, TMB is not certainly positively related to tumour immune response as it failed to predict the response to ICIs in a considerable portion of patients in previous clinical trials. By a machine learning approach in search of biomarkers for immunotherapy implications for HGSOCs, we identified the ten most dominant factors determining the immunogenicity of HGSOCs. Interestingly, we found that BRCA1 mutated tumours presented a potent immunogenic phenotype, independent of TMB, meeting the criteria of both our dominant factors and the determinants of immunogenicity established before. Our findings provide evidence that BRCA1‐mutation may be served as a predictive biomarker in guiding ICI therapies for the patients with HGSOCs.

## 1 INTRODUCTION

Cancer immunotherapy, such as Nivolumab and Pembrolizumab, has been recently identified as a promising treatment across a number of solid tumour types, and its clinic licensing progressively expedited as standard‐of‐care for patients.^1^ However, in comparison with other FDA‐approved tumour types, the observed clinic benefits from immune checkpoint inhibitors (ICIs) for patients with high‐grade serous ovarian cancers (HGSOCs) remain unsatisfying.^2^ In a phase I trial evaluating the anti‐tumour efficacy of Nivolumab, despite limitations of the small cohort size, the objective response rate (ORR) was 15% and disease control rate was 45% in patients with platinum‐resistant ovarian cancer.^3^ In KEYNOTE‐28 trial which exploring the activity of Pembrolizumab in several solid tumours, outcome of ovarian cancer have been disclosed—the ORR was 11.5%, and only 23.1% showed tumour shrinkage from baseline for patients failed to prior chemotherapy.^4^ Such response rate is fuelling an urgent need to develop novel biomarkers that enable the efficient selection of patients with greater likelihood to benefit from the ICI treatment.

Growing evidence support the critical role of immunophenotype of tumour microenvironment (TME) in predicting therapeutic response to immunotherapies. Assessment by immunohistochemistry staining of PD‐L1 expression is a logical biomarker to filter populations with favourable prognosis to anti‐PD‐1 or anti‐PD‐L1 therapies.^5^ Besides the PD‐L1 expression, patients with higher expression of T‐effector interferon‐γ signature,^6^, ^7^ expanded T cell repertoire and higher tumour‐infiltrating lymphocyte density were seen with improved disease control.^8^, ^9^ All of the above characteristics were considered because of the immunogenicity of TME. The immunogenicity was initiated by the somatic mutations in cancer cells, which likely produced a spectrum of tumour‐specific neoantigens to provoke the cytolytic activity. Thus, theoretically, the total mutation burden (TMB) should be able to determine the patients' responsiveness to ICIs. Consistently, ICIs showed to be more effective against tumour types with higher mutation load, such as non‐small cell lung cancer and melanoma, implying a favourable clinical prognosis correlated with an increased capacity to generate neoantigens for immune eradication. However, for patients with HGSOCs, whether the TMB is in parallel with their immunogenicity and what aspects in TME determine the immunogenicity in specific remain less clear.

Here, we aimed to characterize the tumour immune microenvironment of HGSOCs by analysing public accessible data on The Cancer Genome Atlas (TCGA). By a machine learning approach, a panel of dominant parameters in determining the immunogenicity was identified. Besides, by comparing the presentation of dominant parameters among specific mutations subgroups, BRCA1‐mutated tumours were found with higher levels of potent immunotherapy‐responsive signatures, providing a rational for the ICIs treatment for BRCA1‐mutation carriers with HGSOCs.

## 2 MATERIALS AND METHODS

### 2.1 TCGA dataset

We studied 7 cancer types from The Cancer Genome Atlas project (TCGA) (https://gdc-portal.nci.nih.gov/): BLCA, LUSC, LUAD, KIRC, SKCM, HNSC and HGSOC. RNA sequencing (RNAseq) data normalized were downloaded from TCGA Data Portal (https://gdc-portal.nci.nih.gov/) and log2‐transformed. Total mutational burden was also retrieved from the database. All data acquisition and analysis were accomplished using R (3.2.2) unless mentioned otherwise.

### 2.2 Gene signatures and scoring for infiltration/activity levels with ssGSEA

To determine the degree of immune cell infiltration in tumours, we used a previously described and validated computational technique.^10^ In brief, this method applies expression‐based gene signatures of immune cell populations to analyse their infiltration in individual tumour samples using ssGSEA. ssGSEA computes an overexpression score for a gene signature by comparing the ranks of the gene in the signature with all other genes in the transcriptome. The degree of immune cell infiltration in HGSOC was characterized by running ssGSEA with 24 expression‐based immune gene signatures, comprising 782 genes in total. The immune cell populations identified using this deconvolution approach included cells involved in innate immunity: mast cells, neutrophils, eosinophils, macrophages, plasmacytoid dendritic cells (pDCs), inactivated DCs, activated DCs, CD56^dim^ natural killer cells (NK cells), and CD56^bright^ NK cells; and in adaptive immunity: B cells, follicular helper T cells (T_FH_), Type 1 help T cells (T_h_1), T_h_2, T_h_17, regulatory T cells (Tregs), Tγδ, effector memory T cells, central memory T cells and CD8^+^ T cells. This algorithm has been orthogonally validated in samples studied with immunofluorescence staining, with high rates of concordance.

### 2.3 Random forest classification

A random forest classification approach based on a multitude of decision trees was introduced. 782 parameters were input to separate tumours with high cytolytic activity from those with low cytolytic activity, and during the procedure, to sort these parameters according to their importance in determining the cytolytic activity. After dimensionality‐reduced visualization by Multidimensional scaling (MDS) algorithm, the proximity of every 2 sampling tissues indicated the bio‐similarity of the immunophenotype between them (as shown in Figure 3 A).

## 3 RESULT

### 3.1 Lower TMB, PD‐L1 expression and cytolytic molecules in HGSOCs

**Figure 1:**
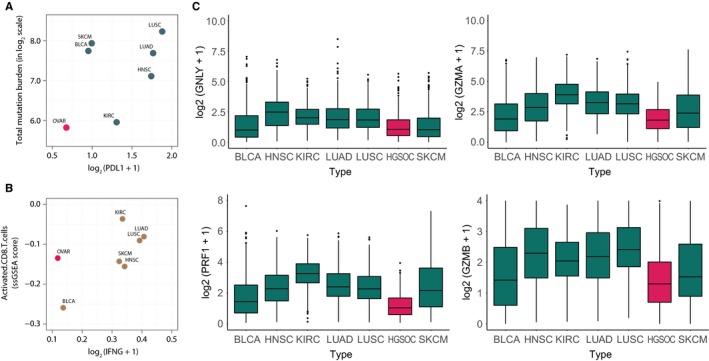
Total mutation load and PD‐L1 expression in several solid tumour types.A, Relative relationships between PD‐L1 expression levels and total mutation burden (TMB) across multiple solid tumour types (BLCA, OVAR, LUAD, NHSC, LUSC, KIRC, SKCM); B, Relative relationships between activated CD8^+^ T cells and interferon‐γ (IFN‐γ) in solid tumours; C, Cytotoxicity represented by GNLY, PRF1, GZMA and GZMB in different tumours

Pre‐existent evidence supported that PD‐L1 expression and TMB could be a manifestation of the responsiveness to ICIs in solid tumours.^11^, ^12^ To evaluate these immune phenotypes of HGSOC, we employed the genomic data of HGSOC from TCGA public database. Genomic data from 6 other solid tumour types, which had been licensed by FDA to receive the anti‐PD‐1/PD‐L1 therapy (SKCM, skin cutaneous melanoma; KIRC, kidney renal clear cell carcinoma; LUSC, squamous cell carcinoma of the lung; LUAD, lung adenocarcinoma; HNSC, head and neck squamous cell carcinoma; BLCA, bladder carcinoma) were also retrieved. As illustrated in Figure 1A, the established predictive biomarkers, both TMB and PD‐L1 expression presented the lowest level in HGSOC among the tumour types studied.

Besides TMB and PD‐L1 expression, the infiltration of cytotoxic T lymphocyte (CTL) and their major mediator interferon‐γ (IFNγ) were also identified enriched in patients responsive to immunotherapy.^13^, ^14^ Thus, we then analysed the relative infiltration level of activated CD8^+^ T cells by the evaluation of expression profiling data as described previously.^15^, ^16^ Similar to the observations of TMB and PD‐L1 expression, the IFN‐γ level remained the lowest in comparison with other tumour types although HGSOC showed a medium level of activated CD8^+^ T cells (Figure 1B).

Several other cytotoxic molecules secreted by CTL were also examined, granulysin (GNLY), perforin1 (PRF1), granzyme A(GZMA) and granzyme B(GZMB). These molecules along with IFN‐γ could indicate the ultimate effector mechanism in cancer immunity cycle and therefore signifying the immunogenicity of the tumour. HGSOC expressed low values consistently in above‐mentioned molecules compared to other tumours (Figure 1C).

The above results demonstrated that either classified by established predictive biomarkers in a variety solid tumour—TMB and PD‐L1 expression, or the potential immune signature of immunotherapy—cytolytic molecules, HGSOC presented the lowest immunogenicity compared to the other 6 types of solid tumour, which have been approved by FDA for the treatment of anti‐PD‐1/PD‐L1 antibodies.

### 3.2 TMB alone could not determine the magnitude of cytolytic immune response

**Figure 2:**
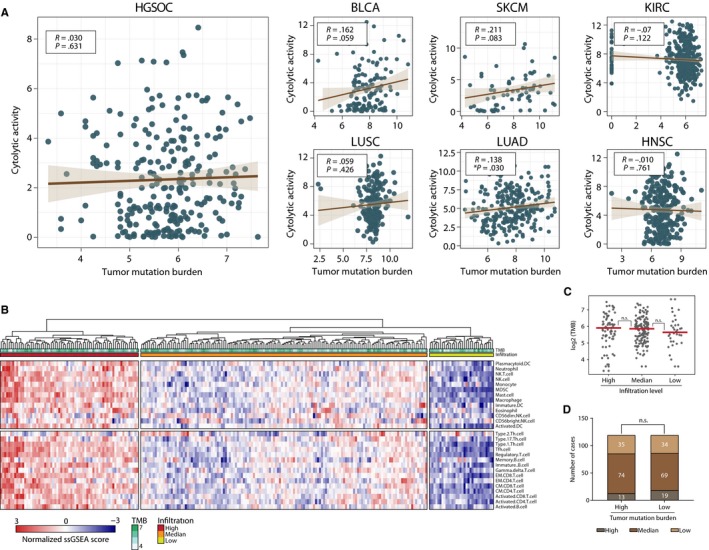
Correlation among total mutation burden (TMB), cytolytic activity and immune cell infiltration.A, Relation between cytolytic activity scores and TMB in multiple solid tumours; B, Heat map demonstrating the infiltration of 28 immune cell types with scores from −3 to 3, coloured blue to red. The status of TMB, mutation of TP53, BRCA1, BRCA2 are also shown; C, TMB in infiltrations high, medium and low clusters; D, Infiltrations of immune cells in TMB high and low groups

Increasing evidence has demonstrated TMB as the predicative biomarkers across a variety of solid tumours, including non‐small‐cell lung cancer (NSCLC) and melanoma.^17^, ^18^ Additionally, patients with deficient mismatch repair (dMMR), as a surrogate of higher TMB, showed more than 50% of ORR in eighteen solid tumour types.^19^ Therefore, we hypothesize that TMB might be the indicator of high immunogenicity for HGSOC as well. To test this, cytolytic activity (CYT) (calculated as the geometric mean of GZMA and PRF1, a scoring system established by Rooney and colleagues^20^) was employed as an indicator for the intensity of cytotoxic immune response. In addition, the correlation between individual TMB and CYT were investigated. Surprisingly, immune responses epitomized by CYT showed a rather slight correlation with TMB across all 7 tumours [HGSOC (*r* = .03, *P* = .631); BLCA (*r* = .162, *P* = .059); LUAD (*r* = .138, *P* = .03); KIRC (*r* = .07, *P* = .122); LUSC (*r* = .059, *P* = .426); HNSC (*r* = 0.01, *P* = .761)] (Figure 2A).

Previous studies have proven that, besides the CYT, immune cell infiltration in tumour microenvironment(TME) is also closely tied with the level of immune activity.^21^ Thus, we analysed the association of TMB and immune cells infiltration. To this end, single‐sample gene set enrichment analysis (ssGSEA) was employed to evaluate the cellular infiltration landscape using RNA‐sequencing data from bulk tissue for individuals, a method which transformed the transcriptomic expression data (TPM) into normalized scores depicting the relative abundance of specific cell types.^10^, ^16^ By an unsupervised algorithm, HGSOC samples can be categorized into 3 clusters based on their spectrum of immune cell infiltration, designated as infiltration high, medium and low (Figure 2B). Coincide with our former observations, TMB level showed no statistic differences among the 3 groups (Figure 2C). Consistently, immune cell infiltrations were neither significantly distinct (Figure 2D) when we divided the samples into TMB High and Low groups (median TMB as cut‐off).

Our above results demonstrated that TMB failed to reflect the immunogenicity of HGSOCs whether inspecting from the magnitude of CYT or immune cells infiltration. These results suggest that TMB might not be a valid predictive biomarker for HGSOC immunotherapies.

### 3.3 Dominant immunological factors in optimizing cytolytic activity beyond TMB

**Figure 3:**
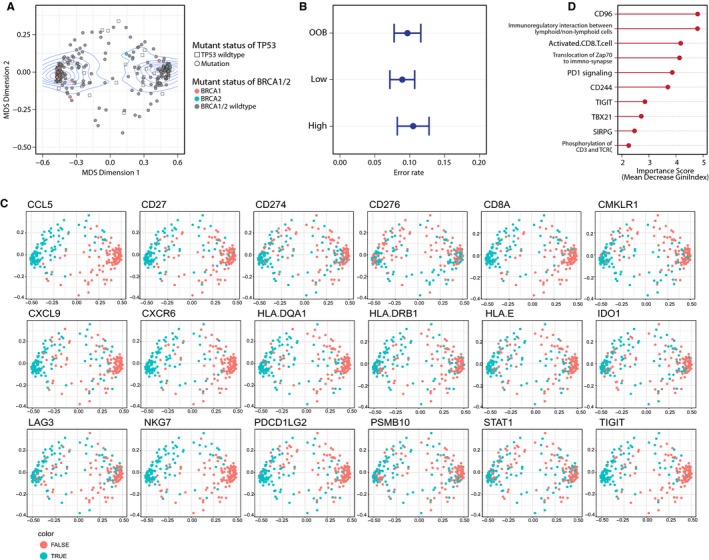
Machine learning identified dominant factors in determining cytolytic activity. A, Density contour Gaussian maximum fitting revealed 2 categories according to levels of cytolytic activity; B, Error rate of group OOB, low and high; C, The ten most important signatures that matters in the categorization; D, Immune features in the 2 categories applied from KEYNOTE‐059 trial. Dots were coloured based on the expression level of each gene

Following the observation that no significant correlation between TMB and CYT in HGSOC, we aim to determine elements that genuinely matters in forming local immune response with a systemic method. A random forest classification approach based on a multitude of decision trees was introduced.^8^ 782 parameters were input to separate tumours with high cytolytic activity from those with low cytolytic activity, and during the procedure, tried to identify the key factors determining the categorization, sorted according to their importance in determining the categorization. After a visualization process, the proximity of every 2 sample tissues in the diagram indicated the bio‐similarity of the immunophenotype between them. Guided by density contour from Gaussian maximum fitting, the analysis revealed 2 distinct categories that predominantly divided on the ground of cytolytic activity (Figure 3A). Out‐of‐bag (OOB) samples providing estimates of model error rate for the decision trees validated the confidence of categories (Figure 3B). To further investigate the phenotypes of above categorization, we applied a panel of 18 genes, which has been validated to predict patients with greater likelihood to respond to immunotherapies in KEYNOTE‐059 trials. Generally, all 18 genes expression levels presented a division in agreement with our categorization (Figure 3C). Higher similarity of categorization were found for genes involved in immune cell identity (CD8A, NKG7 and STAT1), antigen presentation (HLA.DQA1, HLA.DRB1, HLA.E, PSMB10), chemokine and chemokine receptor (CCL5, CMKLR1, CXCL9, CXCR6) and co‐stimulatory/inhibitory checkpoint molecules (CD27, CD274, CD276, IDO1, LAG3, PDCD1LG2, TIGIT) (Figure 3C). These results indicated that the immunophenotype of our machine learning categorization clearly distinguished, in other word, differentiating namely immunological “hot” and “cold” tumour microenvironment.

To identify the most important determinant factors discriminating immunological “hot” or “cold” HGSOCs, we calculated the important score (measured as the mean decrease in accuracy) overall cross‐validated predictions. We found that the top ten vital features are the expression of CD96 molecule, the pathway of immunoregulatory interactions between lymphoid and non‐lymphoid cells, activated CD8^+^ T cell, translocation of Zap70 to immune‐synapse, PD‐1 signalling, molecule CD244, TIGIT, TBX21, SIRPG and phosphorylation of CD3 and T cell receptor (TCR) (Figure 3D).

### 3.4 Higher immunotherapy‐responsive signatures for patients harbouring *BRCA1*‐mutation

**Figure 4:**
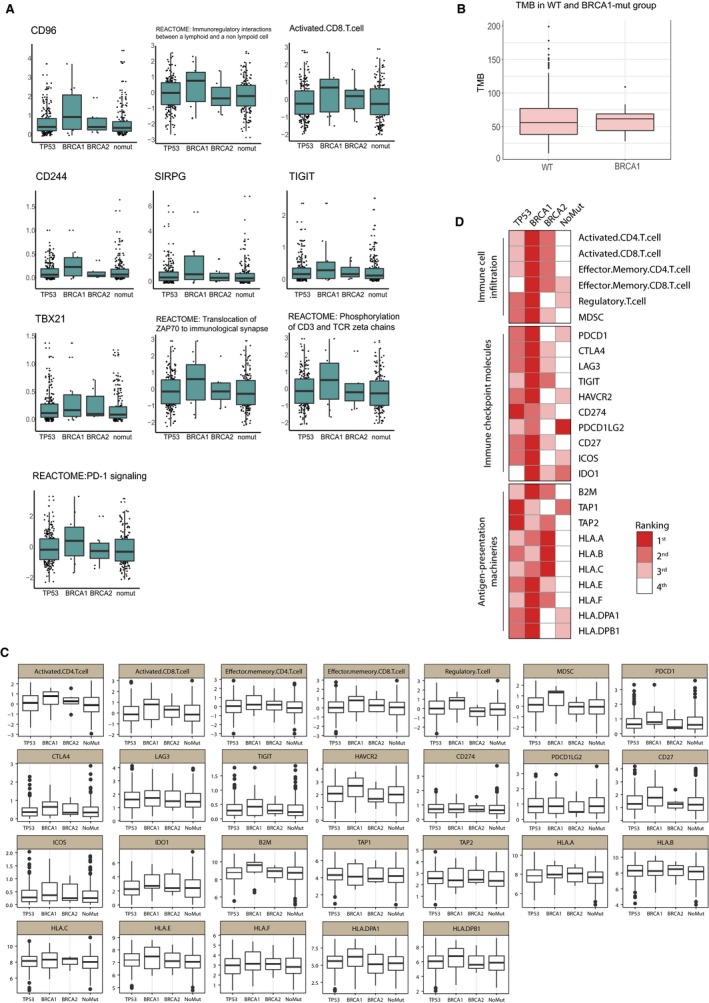
Immunogenicity based on specific somatic mutation site. A, Ten most important factors screened previously in TP53, BRCA1, BRCA2 mutation and non‐mutation groups; B, Total mutation burden (TMB) in WT and BRCA1 mutated groups; C, D, Ranking and scoring of the determinants of immunogenicity in the 4 groups

Specific oncogenic alterations led to substantial different sensitivity to immunotherapies.^22^ Thus, we wondered if certain genomic mutations in HGSOCs could indicate a superior immunogenicity. HGSOCs are characterized by genomic alterations and instability, and DNA copy number abnormalities. In 2011, an analysis of 489 HGSOC samples found that TP53 mutations were presented in 96% of tumours, and homologous repair(HR) gene alterations were shown in about 50% of tumours, including germline mutations in either BRCA1 (9%) or BRCA2 (8%) and also somatic mutations in either one of these 2 genes (3%).^23^, ^24^ To this end, we classified the samples by TP53, BRCA1, BRCA2 abnormalities. Interestingly, the ten crucial factors we screened previously presented higher levels in the BRCA1‐mutated group in comparison with other alterations and wild‐type, which suggested that BRCA1‐mutated HGSOCs may incline to a better immune responsiveness (Figure 4A). And this trend is independent of TMB because TMB showed no difference between WT group and BRCA1‐mutated group (Figure 4B). To confirm this hypothesis, another panel of determinants of immunogenicity in solid cancers were introduced.^8^This panel includes key immune cells and molecules that outline the tumour microenvironment of most solid tumours. Enrichment of these immunological factors was validated to have a better immune response. The results kept accordance with the schema showing the highest level of expression in BRCA1‐mutated samples, only except for TAP1, TAP2, HLA.B (Figure 4C). Relative rankings are shown in Figure 4D. This observation verified our hypothesis that HGSOCs with BRCA1 mutation could be related to a more immunogenic microenvironment.

## 4 DISCUSSION

In the present study, we tried to dig deeper into the unsatisfactory outcome of immunotherapies in HGSOCs through comparison among multiple solid tumour types and reveal more details of the tumour immune microenvironment of HGSOCs. A rather low immunogenicity of HGSOCs was shown as low TMB, low PDL1 in late progression of tumours and low cytotoxic activity. This may be resulted from the genetic characteristics of HGSOCs. Comparing to other solid tumour types, HGSOCs harbours more inactivating mutation in tumour suppressor genes such as p53, NF1, RB1 but rare activating mutations in oncogenes such as KRAS, BRAF, PTEN, HERS, EGFR and KIT.^25^ The facts that ovarian cancers are often discovered in the late course of disease and this research chose high‐grade ovarian cancers as objects may also contribute to the low immunogenicity detected.

Against traditional views, we failed to detect a relationship between TMB and tumour immune response represented by cytolytic activity or immune cell infiltration. This may have several feasible reasons. Firstly, mutations in HGSOC tumours may not be intrinsically more immunogenic or generating neoantigens with appropriate affinity to HLA molecules. Secondly, tumour‐infiltrated T cells may be rejected entering tumour niche or be exhausted regulated by other immune suppressors and checkpoint molecules. In other words, TMB may not serve well as the biomarker for immunotherapies in HGSOCs.

To discover novel biomarkers for ICI therapies, HGSOC samples were classified according to a machine learning approach on the basis of levels of cytolytic activity. Through this method, we also filtrated the top ten key factors that matters in this classification. Top 3 factors are CD96, the pathway of immunoregulatory interactions between lymphoid and non‐lymphoid cells and activated CD8^+^ T cell. CD96 may play a role in the adhesive interactions of activated T cells and NK cells during the late phase of the immune response. It may also function in antigen presentation. Activated CD8^+^ T cell had been proven to be an important prognostic factor in ovarian cancers and many other tumour types.^26^

Interestingly, we found that BRCA1‐mutated tumours expressed a high level of the factors we emphasized in the random forest classification leaning to a more immune‐active phenotype and also showed impressively consistent performance in the panel of determinants of immunogenicity in solid tumours.^8^ And this implication is independent of TMB. To explain the phenomenon, the role of these proteins of BRCA1 or BRCA2 in the maintenance of genome integrity has received the most attention. Both of them are crucial for the process of DNA repair by homologous recombination (HR).^27^

That leads to the discussion of the association between BRCA mutations and immunity, which is under investigated at present. Tumours with BRCA1 or BRCA2 mutations had increased immune infiltrates compared with high‐grade serous without mutations.^28^ Strickland et al^29^ reported immunohistochemistry studies demonstrating that BRCA1/2‐mutated tumours exhibited significantly increased CD3^+^ and CD8^+^ tumour‐infiltrating lymphocytes (TILs) compared to HR proficient (tumours without alterations in HR genes) tumours. Another investigation also showed the presence of intraepithelial cytotoxic T lymphocyte (CTL) also significantly correlates with loss of BRCA1.^30^ Animal experiments are undergoing mainly focusing on combined therapies.^31^ Recent work in a TP53^−/−^ BRCA1‐mutant murine breast cancer model indicates that double blockade with 2 immune checkpoint inhibitors increases the number of tumour‐infiltrating lymphocytes and overall survival after DNA damaging chemotherapy.^32^ Another animal experiment showed that CTLA‐4 antibody, but not PD‐1/PD‐L1 blockade, synergized therapeutically with the Poly (ADP‐ribose) polymerase (PARP) inhibitor, resulting in immune‐mediated tumour clearance and long‐term survival in the majority.^31^ These findings suggest an approach to enhance the impact of immune checkpoint blockade in BRCA‐mutated tumours.

Reports also demonstrated that BRCA2 mutated tumours are more sensitive to chemotherapies (mainly platinum‐based treatment) rather than BRCA1 mutated tumours.^11^More work in the field of mechanism of BRCA gene mutation altering sensitivity to chemotherapy and immunotherapy should be taken out to guide treatment combination or to specify the rules of patient's selection with more details.

Several next‐generation sequencing techniques have been developed to detect BRCA1 and BRCA2 mutations^33^, ^34^ and thanks to the clinical application of PARP, an effective and reliable detection of these mutations in formalin‐fixed and paraffin embedded (FFPE) tissue samples has recently been developed.^35^ With the development of mutation detecting techniques, the clinical affirmation of our research will be possible although a large cohort will be needed because of the low mutation rate of BRCA in the whole heterogenic colony of ovarian cancer. It is hoped that in the future, our effort could balance the efficient biomarkers tests, comprehensive disease information and maximized patients' benefits.

## CONFLICT OF INTEREST

The authors confirm that there is no conflict of interests.
